# Colorectal Cancer Cells Increase the Production of Short Chain Fatty Acids by *Propionibacterium freudenreichii* Impacting on Cancer Cells Survival

**DOI:** 10.3389/fnut.2018.00044

**Published:** 2018-05-24

**Authors:** Marta R. Casanova, João Azevedo-Silva, Ligia R. Rodrigues, Ana Preto

**Affiliations:** ^1^Centre of Biological Engineering, University of Minho, Braga, Portugal; ^2^Centre of Molecular and Environmental Biology, Department of Biology, University of Minho, Braga, Portugal

**Keywords:** colorectal cancer, *Propionibacterium freudenreichii*, probiotic, short chain fatty acids, acetate, propionate

## Abstract

*Propionibacterium freudenreichii* is a commercially relevant bacterium with probiotic potential. This bacterium can exert protective effects particularly against colorectal cancer (CRC), via the production of short chain fatty acids (SCFA), namely acetate and propionate. In this work, we aimed to evaluate the performance and adaptation capacity of *P. freudenreichii* to a simulated digestive stress using different culture media, namely YEL, Basal medium, Mimicking the Content of the Human Colon medium (MCHC) and DMEM. The effect of the fermented culture broth on CRC cells survival and of CRC cells conditioned media on the bacteria performance was also evaluated. Basal medium was found to be the best for *P. freudenreichii* to produce SCFA. MCHC medium, despite being the medium in which lower amounts of acetate and propionate were produced, showed higher acetate and propionate yields as compared to other media. We also observed that the presence of lactate in CRC cells conditioned growth medium resulting from cell metabolism, leads to an increased production of SCFA by the bacteria. The bacterial fermented broth successfully inhibited CRC cells proliferation and increased cell death. Our results showed for the first time that *P. freudenreichii* performance might be stimulated by extracellular lactate produced by CRC metabolic switch also known as “Warburg effect,” where cancer cells “ferment” glucose into lactate. Additionally, our results suggest that *P. freudenreichii* could be potentially used as a probiotic in CRC prevention at early stages of the carcinogenesis process and might help in CRC therapeutic approaches.

## Introduction

Colorectal cancer (CRC) constitutes a major concern in developed countries being associated to lifestyle which is emerging as a critical element for its prevention (1). Evidences have shown that low fat and high dietary fiber diet could be protective against CRC (2–4). The major impact of diet habits on the prevalence of CRC has triggered the design of an optimal diet regimen and/or the development of food supplements specifically reducing the risk of cancer (5–7). A growing interest on the use of probiotics as cancer-preventing/treating agents has been registered (8, 9). Propionibacteria is an important class of probiotics that is well-known for its long history of consumption in Swiss type cheeses (10). During the cheese making process, *P. freudenreichii* resists the harsh physical and chemical stresses exhibiting a high resistance to the digestive stresses which is essential for its survival in the gut and also for exerting its beneficial effects (11, 12). *P. freudenreichii* health beneficial properties are a result of the broad variety of its functional metabolites, including acetate and propionate (10, 13–15). Short chain fatty acids (SCFA), namely acetate, propionate and butyrate, are naturally present in the human gut. SCFA are required to balance the redox equivalent production in the anaerobic environment of the gut, as well as to maintain the intestinal homeostasis (5, 16). Several studies have showed a variety of biological effects of SCFA, and there is a vast number of reports, some of them from our group, showing new mechanisms underlying the effects of these molecules (17–23). SCFA may have opposing effects either inducing/inhibiting autophagy and hence inhibiting proliferation of cancer cells or inducing apoptosis (24–26). In this sense, our group has recently demonstrated that acetate induces lysosomal membrane permeabilisation and the release of Cathepsin D (22). Moreover, we showed that Cathepsin D protects the CRC cells from acetate-induced apoptosis through autophagy-independent degradation of damaged mitochondria (23). The anti-neoplastic effects of *P. freudenreichii* have been evaluated by several research groups (11, 12, 15, 17, 27, 28). Jan et al. (17) demonstrated that the *Propionibacterium* strains induce cell death in human cancer cell lines, such as HeLa, HT29 and Caco2 cells, apparently *via* the SCFA produced. Lan et al. (28) demonstrated in an *in vivo* study, that *P. freudenreichii* TL133, *via* its metabolites, facilitated the elimination of damaged cells by apoptosis in the rat colon after genotoxic insult and may play a protective role against CRC. Maintaining SCFA in the gut, at levels that protect normal colon mucosa cells and kill CRC cells is of utmost relevance for the CRC prevention and/or therapy. Therefore, our aim was to evaluate the SCFA production capacity of *P. freudenreichii* subsp. *freudenreichii*, as well as to study the bacteria capacity to adapt to the digestive stress. Additionally, we aimed to evaluate the influence of the bacteria medium on CRC cells survival and on the other hand the effect of CRC cells conditioned medium in bacteria performance in order to better understand the crosstalk between *P. freudenreichii* and CRC cells.

## Materials and methods

### Bacterial strains and growth conditions

*Propionibacterium freudenreichii* subsp. *freudenreichii* DSM 20271 was purchased from DSMZ (German collection of microorganisms and cell cultures). Freeze dried bacteria were reactivated and routinely cultivated on yeast extract-lactate (YEL) medium at 37°C without agitation. A *P. freudenreichii* tolerant to a simulated digestive stress (adapted bacteria) was also used. *P. freudenreichii* adaptation was performed as described by Lan et al. (12) with some modifications. Briefly, the digestive stress adaptation was performed by exposing the bacteria to pH 5.0 (1 h), pH 2.0 (0.5 h) and finally to 0.1% (w/v) bile salts (2 h). The experiment was performed at 37°C without agitation and under anaerobic conditions. The *P. freudenreichii* subjected to a digestive stress challenge was further inoculated in liquid MCHC in order to recover a robust adapted bacterial culture. After growth (up to OD_650nm_ of 0.8–1.0), the adapted bacterial culture was transferred to fresh liquid MCHC media to reactivate its metabolism and was characterized regarding growth and SCFAs production in different media.

Cultures of normal and adapted *P. freudenreichii* were grown under anaerobic conditions at 37°C without agitation and monitored at defined time points (0, 16, 24, 40, 48, 64, and 72 h) by measuring the absorbance of cell suspensions at 650 nm in a spectrophotometer (Hach, Germany). The culture media used to grow the bacteria were: YEL medium (tryptone, 10 g L^−1^; sodium acetate, 8.4 g L^−1^; yeast extract, 5 g L^−1^; resazurine 0.5 mg L^−1^; KH_2_PO_4_, 0.5 g L^−1^; Na_2_HPO_4_.H_2_O, 0.5 g L^−1^; 1 mL L^−1^ of trace element H^+^ solution; 1 mL L^−1^ of trace element OH^−^ solution; 50 mL L^−1^ of salts and vitamins solution; 50 mL L^−1^ of bicarbonate solution). Trace elements H^+^ solution contains HCl (1.8 g L^−1^), H_3_BO_3_ (61.8 mg L^−1^), MnCl_2_ (61.3 mg L^−1^), FeCl_2_ (1.0 g L^−1^), CoCl_2_ (64.5 mg L^−1^), NiCl_2_ (12.9 mg L^−1^), and ZnCl_2_ (67.7 mg L^−1^). Trace elements OH^−^ solution contains NaOH (0.4 g L^−1^), Na_2_SeO_3_ (17.3 mg L^−1^), Na_2_WO_4_ (29.4 mg L^−1^), and Na_2_MoO_4_ (20.5 mg L^−1^). The salts and vitamins solution contains NH_4_Cl (24 g L^−1^), NaCl (24 g L^−1^), MgCl_2_.6H_2_O (8 g L^−1^), CaCl_2_.2H_2_O (8.8 g L^−1^), biotin (20 mg L^−1^), nicotamid (0.2 g L^−1^), p-aminobenzoic acid (0.1 g L^−1^), thiamin (0.2 g L^−1^), panthotenic acid (0.1 g L^−1^), pyridoxamine (0.5 g L^−1^), cyanocobalamine (0.1 g L^−1^), riboflavin (0.1 g L^−1^), folate (50 mg L^−1^), and lipoate (50 mg L^−1^). The bicarbonate solution contains NaHCO_3_ (80 g L^−1^), and Na_2_S.9H_2_O (5 g L^−1^). Basal medium (BM) is similar to YEL medium but without tryptone and containing 1 g L^−1^ of yeast extract. The medium “mimicking the content of the human colon” (MCHC) (29) is composed by pectin (0.5 g L^−1^), xylan (0.5 g L^−1^), mucin (0.5 g L^−1^), starch (0.5 g L^−1^), peptone (0.5 g L^−1^), tryptone (2.5 g L^−1^), yeast extract (0.5 g L^−1^), bile salts (0.05 g L^−1^), K_2_HPO_4_ (2.0 g L^−1^), NaHCO_3_ (0.2 g L^−1^), NaCl (4.5 g L^−1^), MgSO_4_.7H_2_O (0.5 g L^−1^), CaCl_2_.2H_2_O (0.45 g L^−1^), MnCl_2_ (0.2 g L^−1^), haemin (50 mg L^−1^), FeSO_4_.7H_2_O (5.0 mg L^−1^), CoCl_2_.6H_2_O (50 mg L^−1^), tween 80 (2.0 mL L^−1^), sodium lactate (2.3 g L^−1^), thiamine HCl (4.0 μg L^−1^), calcium pantothenate (10 μg L^−1^), nicorinic acid (5.0 μg L^−1^), p-aminobenzoic acid (5.0 μg L^−1^), biotin (2.0 μg L^−1^), vitamin B_12_ (0.5 μg L^−1^), and cysteine (0.8 g L^−1^). Dulbecco‘s Modified Eagle‘s Medium (DMEM; Biowest, France) supplemented with 10% (v/v) heat inactivated fetal bovine serum (FBS; Biowest, France) was also used to grow bacteria cultures. Bacteria were also cultured in CRC cells conditioned medium which consists in the supernatants collected after CRC cells growth. CRC cells were cultivated in 75 cm^2^ tissue culture flasks (TPP, German) at a density of 20 × 10^4^ cell mL^−1^, using DMEM medium without antibiotic. The CRC conditioned medium was collected after 72 h of incubation and was then diluted (1:1) in fresh DMEM medium.

### Analysis of small chain fatty acids, glucose, and biomass

Lactate, acetate and propionate were analyzed by high-performance liquid chromatography (HPLC) equipped with an UV detector (Jasco, Spain) using a Metacarb 67H column (Varian, USA) operated at 60°C with 0.01 N H_2_SO_4_ as the mobile phase at an 0.6 mL min^−1^ flow rate. Glucose concentration was measured using the same HPLC system but equipped with a Metacarb 87H column (Varian, USA) and a RI detector (KNAUER, German) operated using the same conditions of temperature and mobile phase and a flow rate of 0.7 mL min^−1^. Samples for further analysis were centrifuged at 10 000 g for 5 min (Eppendorf, Spain), filtered with 0.2 μm filters (GE Healthcare Life Science, Germany). Standard solutions of acetate, propionate and glucose were used in a wide range of concentrations (0–10 g L^−1^) was used to elaborate the calibration curves. An area of “x” represents (0.0034x −0.0027; *R*^2^ = 0.9988), (0.0032x −0.0027; *R*^2^ = 0.9991) and (0.0026x + 36.1222; *R*^2^ = 0.9992) g L^−1^ (for x > 0) of acetate, propionate and glucose, respectively.

Biomass was determined from a standard curve of optical density vs. dry weight. Dry weights for the standard curve were obtained by filtering the culture through 0.2 μm filters. After filtration, the filters were dried and weighed. The standard curve was plotted from the mean values of two determinations, and the biomass was obtained from the least-squares regression line. All fermentations were performed in triplicated, except the DMEM fermentation which was performed in duplicated.

### CRC cells culture conditions

The human CRC derived cell line RKO (CRL-2577) was kindly provided by IPATIMUP. Cells were grown as monolayers at 37°C in a humidified incubator with 5% CO_2_, in DMEM medium supplemented with 10% (v/v) FBS and 1% (v/v) penicillin-streptomycin (Invitrogen, Portugal). RKO cells were kept in exponential growth phase and subcultured once or twice a week. For the assays, subconfluent cells, in exponential growth phase, were detached with trypsin/EDTA (Invitrogen, Portugal) and ressuspended in fresh medium at the appropriate density.

### CRC cells proliferation assay

In all treatments performed in this work, cells in exponential growth phase were cultured in 24-well plates at a cellular density of 1 × 10^5^ cell mL^−1^ in a final volume of 0.5 ml per well, excluding the cell cycle analysis in which cells were cultured in 6-well plates at a cellular density of 1.5 × 10^5^ cell mL^−1^ in a final volume of 1.5 mL per well. Cells were incubated at 37°C in a 5% CO_2_ atmosphere during 24 h, to allow cell adhesion. After adhesion, the culture medium was removed and replaced by different treatment conditions.

To evaluate the effect of pure acetate (sodium acetate; pH 7.1–7.4) and propionate (sodium propionate; pH 7.1–7.4) on CRC cells proliferation, cells were treated with the pure acids, acetate and propionate, at different concentrations either alone or combined. The half maximal inhibitory concentration (IC_50_) (6.6 g L^−1^; 4.4 g L^−1^) and 30% maximal inhibitory concentration (IC_30_) (4.4 g L^−1^; 2.9 g L^−1^) of acetate and propionate [concentrations previously determined in our lab (22)], respectively, were used. Pure acids at the same concentration of acetate (0.79 g L^−1^) and propionate (2.6 g L^−1^) that were detected in the DMEM fermented broth were used to treat CRC cells for 48 h. Hydrogen peroxide (1 mM) was used as a positive control, while fresh DMEM medium was used as negative control. Both control media conditions have in their composition 1% (v/v) penicillin-streptomycin. The absence of Mycoplasm contamination was regularly evaluated in the cell lines according to the laboratory regulation.

To evaluate the effect of the SCFA produced by adapted and normal (non-adapted) *P. freudenreichii* on the CRC cells, cells were treated with BM and DMEM fermented broths by adapted and normal bacteria diluted (1:1) in fresh DMEM medium. Fermented broths were collected after bacterial growth through centrifugation at 10,000 g for 20 min (HERAEUS, Germany) and filtration using sterile filters of 0.2 μm. The fermented broth pH was adjusted to 7.10−7.40 and kept at −20°C with parafilm. A part of the fermented broth was sterilized (121°C; 15 min; 1 bar) to obtain deproteinized fermented broth. Hydrogen peroxide (1 mM) was used as a positive control; DMEM medium diluted (1:1) in sterile BM medium as negative control for the BM fermented broth and DMEM medium diluted (1:1) in CRC cells conditioned medium as a negative control for the DMEM fermented broth. CRC cells conditioned medium used in this context was a DMEM medium depleted of nutrients. Tissue culture flask of 25 cm^2^ containing CRC cultures were maintained for a week without replacing the culture media, assuring that all the nutrients in the medium were consumed. After 1 week in culture, the RKO conditioned medium was removed to a 15 mL falcon and the supernatant was collected through centrifugation at 1,000 g for 10 min. Controls containing the pure SCFA at the same concentrations present in fermented broths were also used.

The sulforhodamine B (SRB) assay was used to access cell proliferation. After treatments, cells were fixed in ice-cold methanol containing 1% acetate, and incubated with SRB for 1.5 h at 37°C. After washing with 1% acetate, the SRB was solubilized (10 mM Tris, pH 10) and absorbance was read at 540 nm in a microplate reader (Molecular Devices, USA). Results were expressed relatively to the negative control, which was considered as 100% of cell proliferation.

### Cell cycle analysis

After 48 h of treatment, cells were harvested by scraping and the medium was collected and centrifuged at 500 g for 3 min. The pellet was resuspended in 500 μL PBS 1x and incubated on ice for 15 min. After this period, 1.5 ml of 96% (v/v) cold ethanol (stored at −20°C) was added to the pellet and was incubated on ice during 15 min, to allow cell fixation. Cells were then washed with 4 ml PBS 1x, centrifuged at 500 g for 3 min at 4°C and the pellet again washed with PBS 1x. The final pellet was resuspended in 500 μL PBS 1x and was incubated with 50 μL of RNase A solution [200 μg mL^−1^ in sodium citrate 1% (w/v)] at 37°C for 15 min. After incubation, 50 μL propidium iodide (PI) staining solution [0.5 mg mL^−1^ in sodium citrate 1% (w/v)] was added and the cells were mixed in a vortex and were incubated at room temperature for 30 min in the dark. Cells with red fluorescence [FL-3 channel (488/620 nm)] were analyzed in Epics XL flow cytometer (Beckman Coulter, USA) with an average of 20,000 counts per sample. Data were analyzed with the Flowing software program (Perttu Terho, Finland) to generate DNA content frequency histograms and quantify the amount of cells in the individual cell-cycle, including sub-G1 population assumed as death cells.

### Statistical analysis

Results correspond to the mean ± standard error of the mean (SEM) of at least three independent experiments. The data were analyzed using an analysis of variance (one-way ANOVA) followed by Tukey's Test to compare all the experiments or Dunnett‘s test to assess the differences between control and different conditions tested. The confidence interval used was 95% and *p* ≤ 0.05 were regarded as statistically significant. All statistical tests were performed using the software Graphpad Prism 5.

## Results

### Production of short chain fatty acids by *P. freudenreichii*

**Figure 1:**
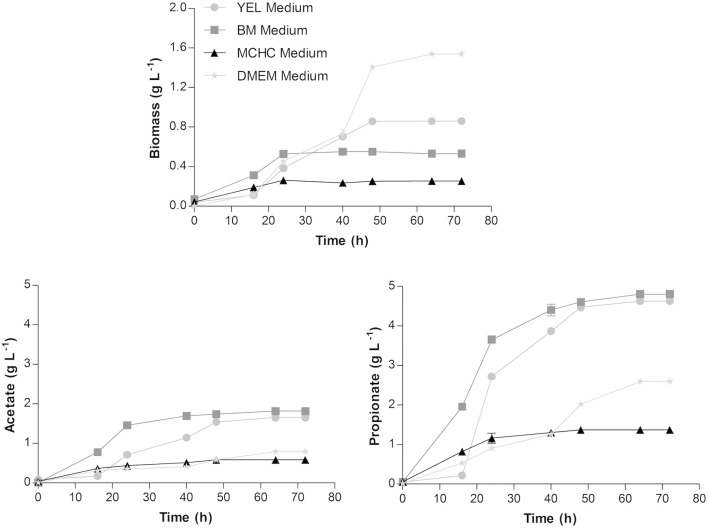
Biomass, acetate and propionate production profiles by *Propionibacterium freudenreichii* in YEL, BM, MCHC, and DMEM media, at 37°C. Each condition was run in triplicate and the results correspond to the mean ± standard error of the mean.

*Propionibacterium freudenreichii* was grown and characterized regarding biomass and SCFA production in different culture media. The results obtained are illustrated in Figure 1.

Biomass production reached ~0.86 ± 0.02; 0.53 ± 0.01; 0.25 ± 0.01, and 1.55 ± 0.01 g L^−1^ in YEL, BM, MCHC, and DMEM media, respectively. The acetate production was found to be around 1.65 ± 0.01; 1.81 ± 0.01; 0.58 ± 0.02, and 0.79 ± 0.02 g L^−1^ in YEL, BM, MCHC, and DMEM media, respectively; while the propionate production was around 4.69 ± 0.01; 4.81 ± 0.02; 1.37 ± 0.03, and 2.60 ± 0.05 g L^−1^ in YEL, BM, MCHC, and DMEM media, respectively. These results showed that higher amounts of biomass production were not correlated with higher amounts of SCFA production. For instance, in DMEM, the bacteria exhibited higher amounts of biomass production; however, lower amounts of SCFA were produced. In MCHC, the bacteria displayed lower amounts of both biomass and SCFA production. The higher production of SCFA was obtained using BM, followed by the YEL medium.

Growth rates, yields and productivities were determined for all culture media tested and are summarized in Table 1. Bacteria grown in DMEM exhibited the highest biomass production when compared to the other culture media evaluated, but showed lower acetate and propionate productivities. Although the lower amounts of acetate and propionate production by *P. freudenreichii* were obtained in MCHC medium, the acetate and propionate yields are high as compared to other media.

**Table 1:** Kinetics of the fermentations conducted with non-adapted and adapted (digestive stress challenged bacteria) *Propionibacterium freudenreichii*.

		**Non-adapted** ***P. freudenreichii***				**Adapted** ***P. freudenreichii***			
		**YEL**	**BM**	**MCHC**	**DMEM**	**YEL**	**BM**	**MCHC**	**DMEM**
	Biomass (g L^−1^)	0.86 ± 0.02	0.53 ± 0.01	0.25 ± 0.01	1.55 ± 0.01	0.80 ± 0.02	0.53 ± 0.03	0.39 ± 0.01^***^	1.67 ± 0.01^***^
	Growth rate (h^−1^)	0.08 ± 0.00	0.07 ± 0.00	0.04 ± 0.00	0.09 ± 0.01	0.11 ± 0.00^***^	0.06 ± 0.01	0.05 ± 0.00	0.08 ± 0.00
Acetate	Concentration (g L^−1^)	1.65 ± 0.01	1.81 ± 0.01	0.58 ± 0.02	0.79 ± 0.02	1.53 ± 0.07	1.11 ± 0.03^***^	0.61 ± 0.02	0.73 ± 0.01
	Yield (g g^−1^)	0.18 ± 0.00	0.21 ± 0.01	0.22 ± 0.01	0.18 ± 0.00	0.18 ± 0.01	0.13 ± 0.00^**^	0.23 ± 0.01	0.18 ± 0.00
	Productivity (g L^−1^ h^−1^)	0.03 ± 0.00	0.04 ± 0.00	0.01 ± 0.00	0.01 ± 0.00	0.03 ± 0.00	0.02 ± 0.00^***^	0.01 ± 0.00	0.01 ± 0.00
Propionate	Concentration (g L^−1^)	4.69 ± 0.01	4.81 ± 0.02	1.37 ± 0.03	2.60 ± 0.05	3.13 ± 0.10^***^	2.91 ± 0.13^***^	1.35 ± 0.05	2.72 ± 0.05
	Yield (g g^−1^)	0.53 ± 0.00	0.55 ± 0.01	0.57 ± 0.01	0.57 ± 0.00	0.37 ± 0.01^***^	0.35 ± 0.02^***^	0.57 ± 0.02	0.66 ± 0.02
	Productivity (g L^−1^ h^−1^)	0.09 ± 0.00	0.10 ± 0.00	0.03 ± 0.00	0.03 ± 0.00	0.07 ± 0.00^***^	0.06 ± 0.00^***^	0.03 ± 0.00	0.04 ± 0.00

Different media were used to culture the adapted and non-adapted bacteria for 72 h at 37°C without agitation. Na-Lactate was the carbon source in all media at an initial concentration of 8.4 g L^−1^ (YEL and BM) and 2.3 g L^−1^ (MCHC), except for the DMEM medium in which glucose was used instead at an initial concentration of 4.1 g L^−1^ Each condition was run in triplicate and the results correspond to the mean ± standard error of the mean. YEL, Yeast extract-lactate medium; BM, Basal medium; MCHC, Mimicking the Content of the Human Colon medium; DMEM, Dulbecco‘s Modified Eagle‘s Medium;

** p < 0.01;

*** *p < 0.001, values significantly different between the same medium*.

### *P. freudenreichii* adaptation to simulated digestive stress

The probiotic efficiency of *P. freudenreichii* depends on its capacity to survive the digestive tract stresses and to remain metabolically active in the gut. In order to obtain bacteria tolerant to this type of stress, a digestive stress challenge was performed. *P. freudenreichii* was exposed to acidic pH for 1.5 h and bile salts concentrations for 2 h, mimicking the digestion process. The performance of adapted *P. freudenreichii* was evaluated and the results obtained are presented in Table 1.

Adapted *P. freudenreichii* displayed an increase in the biomass concentration and growth rate, in DMEM and MCHC media, when compared to the performance of the non-adapted *P. freudenreichii*. However, the production of SCFA, particularly propionate production, were negatively affected by the digestive stress challenge. Significant statistical differences could be observed between YEL and BM medium (*p* < 0.001) when comparing the results obtained for the normal and adapted bacteria (digestive stress challenged bacteria). This effect was also observed for acetate production. Interestingly, the adapted *P. freudenreichii* acetate yields in MCHC media were positively affected by the digestive stress challenge showing a slight increase (Table 1).

### Effect of colorectal cancer extracellular medium on propionibacteria performance

**Figure 2:**
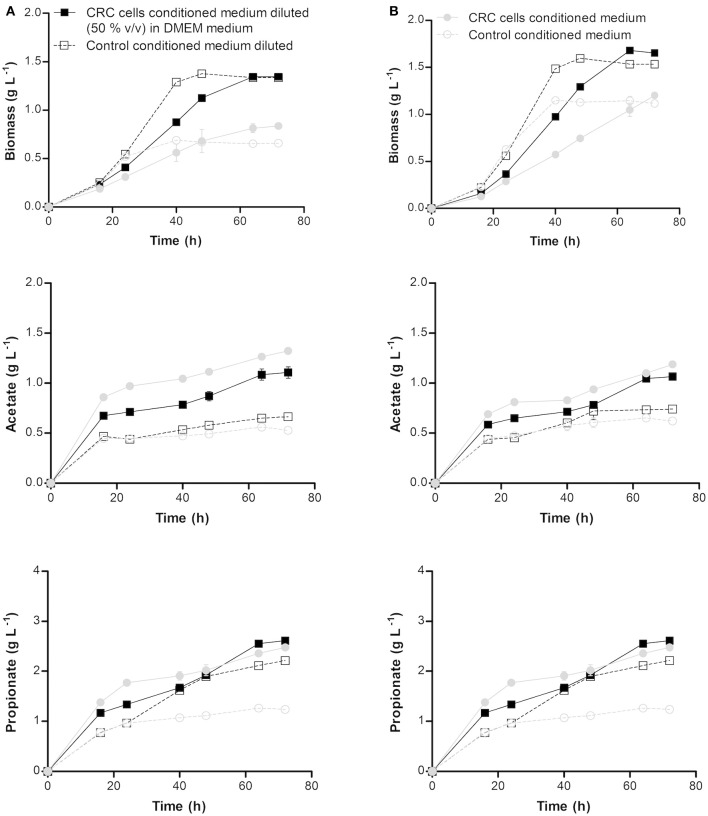
Biomass, acetate and propionate production profiles by non-adapted **(A)** and adapted **(B)** *Propionibacterium freudenreichii* at pH 7.0 and 37°C in non-diluted and diluted [with fresh DMEM (1:1)] colorectal cancer cells conditioned medium and the respective controls. Conditioned medium consists in the supernatants collected after colorectal cancer cells growth in a DMEM medium without antibiotic for 72 h at 37°C in a humidified incubator with 5% CO_2_. Each condition was run in duplicate and the results are represented by the mean ± standard error of the mean.

The effect of co-culture of CRC cells with probiotic bacteria in the production of SCFA in normal and adapted *P. freudenreichii* was studied through simulation using medium where CRC cells were grown (conditioned medium). The diluted and non-diluted conditioned medium containing 2.7 and 1 g L^−1^ of glucose, respectively, were used to simulate the effect of CRC cells in the growth of normal (non-adapted) *P. freudenreichii* and on SCFAs production. Diluted and non-diluted conditioned medium containing 3.2 and 1.8 g L^−1^ of glucose, respectively, were used with the same purpose for the adapted bacteria. As controls, fresh DMEM medium with the same concentrations of glucose present in the conditioned medium were used. The results presented in Figure 2 showed that bacteria growth was negatively affected by CRC cells conditioned medium (simulating its indirect contact). A slow growth with a long stationary phase was observed, although the final biomass concentration was similar to the control. Adapted and non-adapted *P. freudenreichii* growth in conditioned medium exhibited similar behaviors. Growth and SCFA production parameters are summarized in Table 2. We could observe statistically significant differences for bacteria grown in conditioned medium comparing to the controls. Yield and productivity parameters of acetate and propionate show also statistically significant increase between conditioned medium and the respective controls. Overall, the conditioned medium led to a better bacteria performance regarding the production of SCFA.

**Table 2:** Kinetics of the fermentations conducted with adapted and non-adapted *Propionibacterium freudenreichii*.

		**Non-adapted** ***P. freudenreichii***				**Adapted** ***P. freudenreichii***			
		**CCM$\frac{1}{2}$**	**CM$\frac{1}{2}$**	**CCM**	**CM**	**CCM$\frac{1}{2}$**	**CM$\frac{1}{2}$**	**CCM**	**CM**
	Biomass (g L^−1^)	1.34 ± 0.01	1.35 ± 0.04^ns^	0.66 ± 0.02	0.84 ± 0.03^*^	1.53 ± 0.00	1.65 ± 0.02^ns^	1.14 ± 0.05	1.20 ± 0.02^ns^
	Growth rate (h^−1^)	0.10 ± 0.01	0.07 ± 0.00^*^	0.10 ± 0.00	0.04 ± 0.00^***^	0.11 ± 0.00	0.10 ± 0.01^ns^	0.13 ± 0.00	0.04 ± 0.00^***^
Acetate	Concentration (g L^−1^)	0.66 ± 0.01	1.11 ± 0.06^**^	0.53 ± 0.02	1.32 ± 0.02^***^	0.74 ± 0.00	1.07 ± 0.00^***^	0.62 ± 0.00	1.19 ± 0.02^***^
	Yield (g g^−1^)	0.24 ± 0.01	0.40 ± 0.02^***^	0.56 ± 0.01	1.26 ± 0.01^***^	0.23 ± 0.00	0.33 ± 0.00^**^	0.36 ± 0.02	0.66 ± 0.01^***^
	Productivity (g L^−1^ h^−1^)	0.01 ± 0.00	0.02 ± 0.00^ns^	0.009 ± 0.000	0.02 ± 0.00^***^	0.01 ± 0.00	0.02 ± 0.00^ns^	0.011 ± 0.001	0.02 ± 0.00^***^
Propionate	Concentration (g L^−1^)	2.21 ± 0.02	2.62 ± 0.06^**^	1.24 ± 0.05	2.48 ± 0.00^***^	2.66 ± 0.02	2.92 ± 0.07^ns^	1.84 ± 0.05	2.53 ± 0.03^**^
	Yield (g g^−1^)	0.78 ± 0.03	0.95 ± 0.02^***^	1.3 ± 0.0	2.36 ± 0.06^***^	0.82 ± 0.01	0.90 ± 0.02^**^	1.00 ± 0.01	1.40 ± 0.01^***^
	Productivity (g L^−1^ h^−1^)	0.03 ± 0.00	0.04 ± 0.00^**^	0.020 ± 0.001	0.04 ± 0.00^***^	0.04 ± 0.00	0.05 ± 0.00^ns^	0.03 ± 0.00	0.04 ± 0.00^**^

*Colorectal cancer cells conditioned medium was used to culture the non-adapted and adapted bacteria. It consists of the supernatants collected after colorectal cancer cells growth in a DMEM medium without antibiotic for 72 h at 37°C in a humidified incubator with 5% CO_2_. Glucose was the carbon source present in this medium. Each condition was run in duplicate and the results correspond to the mean ± standard error of the mean*.

* p < 0.05;

** p < 0.01;

*** *p < 0.001, values significantly different between conditioned medium conditions and respective controls; “ns” corresponds to non-significant differences. CM$\frac{1}{2}$, colorectal cancer cells conditioned medium diluted (1:1) in fresh DMEM; CCM$\frac{1}{2}$, control of diluted colorectal cancer cells conditioned medium; CM, Colorectal cancer cells conditioned medium; CCM, control of colorectal cancer cells conditioned medium*.

The increased SCFA production in conditioned medium could be due to the presence of some metabolites produced by CRC cells that are positively assimilated by bacteria. In order to identify such compounds, present in conditioned medium which could explain the improved SCFA production, CRC cells conditioned medium diluted and non-diluted with fresh DMEM medium were analyzed by HPLC.

The HPLC analyzed showed that lactate was detected in the conditioned medium but not in the controls, what is in accordance to the production of lactate by CRC cells metabolism switch, also known as “warbourg effect” where cancer cells “ferment” glucose into lactate. Non-diluted and diluted conditioned medium used to culture normal *P. freudenreichii* was composed of 2.4 and 0.9 g L^−1^ of lactate, respectively, while 1.6 and 0.8 g L^−1^ of lactate was quantified in non-diluted and diluted conditioned medium used to culture adapted *P. freudenreichii*. The presence of lactate in CRC cells conditioned medium diluted and non-diluted with fresh DMEM can explain the improvement of the biotransformation performance by probiotic bacteria given that lactate is the favorite carbon source for these bacteria.

### Effect of short chain fatty acids produced by propionibacteria on colorectal cancer cells proliferation

**Figure 3:**
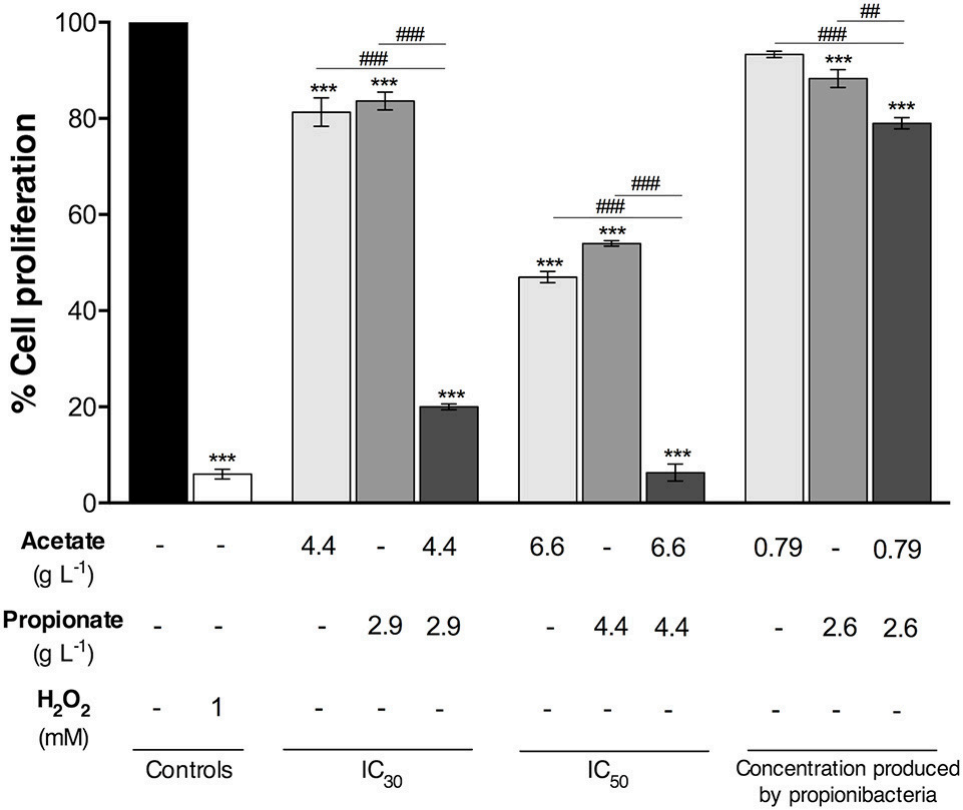
Proliferation of colorectal cancer cells treated with pure acetate and propionate either alone or combined. Cells were incubated for 48 h with IC_50_, IC_30_ and the acetate and propionate concentrations similar to the ones that were determined in the *Propionibacterium freudenreichii* fermented broth. Fresh DMEM medium and hydrogen peroxide (1 mM) were used as a negative and positive control, respectively. Values represent the mean ± standard error of the mean of at least three independent experiments.^***^p < 0.001, compared with negative control cells. ^##^*p* < 0.01, ^###^*p* < 0.001, comparing between condition.

In order to evaluate the effect of acetate and propionate produced by bacteria on CRC cell proliferation by SRB assay, cells were treated with concentrations similar to those obtained in the bacteria medium. The IC_50_ and IC_30_ values of acetate (4.4; 6.6 g L^−1^) and propionate (2.9; 4.4 g L^−1^) alone were previously determined in our laboratory (22). As a comparison, the cells were incubated with acetate or/and propionate (between pH 7.1–7.4) at the IC_50_ and IC_30_ concentrations for 48 h (Figure 3).

The IC_50_ concentration of acetate and propionate reduced cell proliferation by ~53 and 46%, respectively. IC_30_ of acetate and propionate led to a reduction in cell proliferation of 19 and 17%, respectively. An acetate concentration of 0.79 g l^−1^ led to a cell proliferation decrease of 7% with no statistical differences when compared to the negative control. A propionate concentration similar to that detected in DMEM fermented broth by *P. freudenreichii* (2.6 g L^−1^) led to a decrease of 12% in cell proliferation when compared with the negative control. The combination of acetate and propionate exhibited a higher effect in cell proliferation. The IC_50_ of acetate and propionate together inhibited cell proliferation almost completely. The IC_30_ of acetate and propionate together reduced cell proliferation by ~80%, being higher than the effect of the IC30 of the acids alone. Proliferation of CRC cells incubated with 0.79 g L^−1^ of pure acetate and 2.6 g L^−1^ of pure propionate (i.e. the SCFA concentrations present in DMEM fermented broth by *P. freudenreichii*) was reduced by ~21% being significantly different from the negative control.

**Figure 4:**
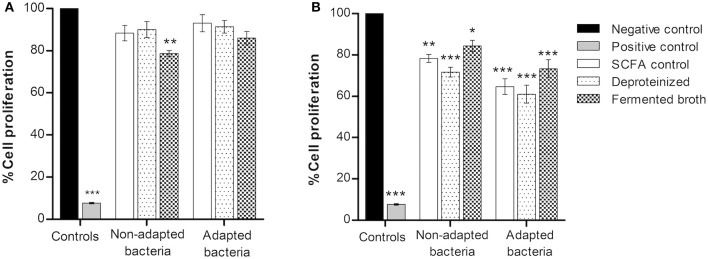
Proliferation of colorectal cancer cells treated with BM **(A)** and DMEM **(B)** fermented broth by adapted and normal *Propionibacterium freudenreichii*. Fermented broths were deproteinized to eliminate any possible cytotoxic effect from contaminating proteins. Pure SCFA at concentrations present on the fermented broth of non-adapted bacteria (0.9 g L^−1^ acetate; 2.4 g L^−1^ propionate) or adapted bacteria (0.6 g L^−1^ acetate; 1.5 g L^−1^ propionate) were used as a SCFA control. Cells were incubated for 48 h with BM and DMEM fermented broths and its respective controls. BM diluted in fresh DMEM medium and CRC cells conditioned medium diluted in fresh DMEM medium were used as a negative control of BM and DMEM fermented broths, respectively, and Hydrogen peroxide (1 mM) as a positive control. Values represent the mean ± standard error of the mean of at least three independent experiments. ^*^p < 0.05; ^**^p < 0.01; ^***^p < 0.001 compared with negative control cells.

In order to evaluate the effect of the SCFA produced by adapted and normal *P. freudenreichii* on the CRC cells, the influence of the fermented broth was also analyzed. Among the four fermented broths used, BM and DMEM were selected since they showed no toxic effect to the CRC cells (*data not shown*). BM diluted in fresh DMEM medium and CRC cells conditioned medium diluted in fresh DMEM medium were used as a negative control of BM and DMEM fermented broths, respectively. Fermented broths were deproteinized (121°C; 15 min; 1 bar) to eliminate any possible cytotoxic effect from contaminating proteins. Diluted fermented broths contained 0.9 g L^−1^ of acetate and 2.4 g L^−1^ of propionate for *P. freudenreichii* and fermented broth of adapted *P. freudenreichii*, containing 0.6 g L^−1^ of acetate and 1.5 g L^−1^ of propionate were used to treat colorectal cancer cells. Cells were also treated with pure SCFA at the concentrations previous described (SCFA control). Results of BM and DMEM fermented broth by adapted and normal bacteria and the respective controls are represented in Figure 4.

Inhibition of CRC cells proliferation was observed only with a statistical significance (*p* < 0.01) in BM fermented broth from normal *P. freudenreichii*. All conditions of DMEM fermented broth (Figure 4B) showed an inhibition of cell proliferation around 10–20%. No statistically significant differences were found between BM fermented broth, BM fermented broth deproteinized and the control with pure SCFA, indicating that the effect of the fermented broths on CRC cells proliferation is due to the bacterial SCFA cytotoxicity.

**Figure 5:**
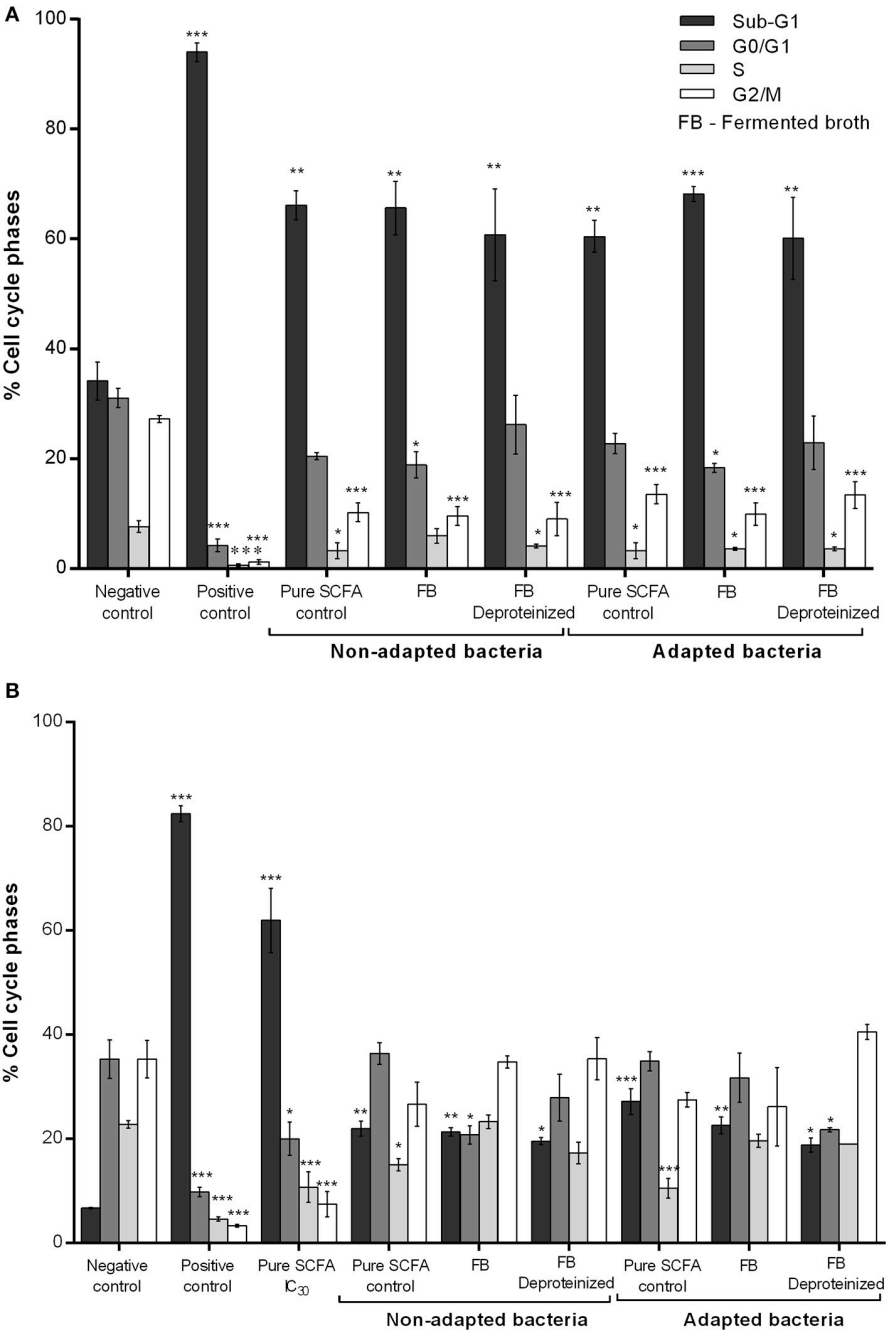
Effect of the BM **(A)** and DMEM **(B)** fermented broth on cell cycle distribution. Analysis of the cell-cycle phase's distribution: G0/G1; S; G2/M and SUB-G1 in CRC cells treated with fermented broth from adapted and non-adapted *P. freudenreichii* and the respective controls. Fermented broths were deproteinized to eliminate any possible cytotoxic effect from contaminating proteins. BM diluted in fresh DMEM medium and CRC cells conditioned medium diluted in fresh DMEM medium were used as a negative control of BM and DMEM fermented broths, respectively. Hydrogen peroxide (1 mM) was used as a positive control. Pure SCFA at concentrations present on the fermented broth of non-adapted bacteria (0.9 g L^−1^ acetate; 2.4 g L^−1^ propionate) or adapted bacteria (0.6 g L^−1^ acetate; 1.5 g L^−1^ propionate) were used as a SCFA control. Values represent the mean ± standard error of the mean of at least three independent experiments. ^*^p < 0.05; ^**^p < 0.01; ^***^p < 0.001, comparing with negative control.

Cell cycle distribution of CRC treated with fermented broths (BM and DMEM) and the respective controls was also studied by flow cytometry measurement of the DNA content of cells stained with PI (Figure 5). SCFA produced by normal and adapted *P. freudenreichii* caused an accumulation of CRC cells in the sub-G1 phase and a decrease in S and G2/M phases, being significantly different compared to the negative control. Moreover, higher accumulation of CRC cells in sub-G1 phase (50–70%) was observed when cells were treated with BM fermented broth than when cells were treated with DMEM fermented broth (20–35%). Cells treated with DMEM fermented broth also seem to be arrested in the G2/M phase of the cell-cycle (10–30%).

## Discussion

Colorectal cancer is a leading cause of mortality in Europe (30), and its occurrence is commonly attributed to the transformation of normal colonic epithelium to adenomatous polyps and ultimately invasive cancer (1). Environmental factors, namely diet and lifestyle, have been reported in epidemiological studies as responsible for the increase in CRC incidence (2–4, 6). SCFA, particularly propionate and acetate, are known to have pro-apoptotic effects (31, 32). These SCFA are the end product of fermentation by physiological probiotic bacteria, such as *P. freudenreichii*, which are present in several dairy products frequently consumed (10, 33). A diet efficient in enhancing the colonic content of SCFA may help to prevent or treat CRC, as a preventive diet or as a complement to cancer therapy, respectively.

In a previous report, our group have shown that acetate *per se* inhibits proliferation and induces apoptosis, leading to lysosomal membrane permeabilization with Cathepsin D release from the lysosome in CRC cells (22). Likewise, we showed that the CRC cells are protected by Cathepsin D from acetate-induced apoptosis through autophagy-independent degradation of damaged mitochondria (23). These observations suggest that propionate and acetate, metabolites from propionibacteria could make it a potential nutraceutical against CRC acting as a probiotic, and pointing to a useful role of SCFA as powerful agents for CRC prevention or therapy.

Although propionibacteria can be seen as a probiotic agent, adaptation to the colonic lumen environment is critical to confirm its potential. It is important that propionibacteria survive and still produce the beneficial metabolites under stress conditions such as host defense mechanisms including pH variations, peristaltism, antimicrobial peptides, and bile acids (34). The competition with resident microbiota for nutrient acquisition and for growth niches needs also to be taking in account (35). However, the influence of this digestive stresses on the production of acetate and propionate have never been investigated. In this work we aimed at study the capacity of propionibacterium to adapt under the digestive stress conditions and to evaluate its anti-cancer capacity.

*Propionibacterium freudenreichii* has a great potential regarding the production of acetate and propionate as end-products, although with different performances when grown in different media. Results of SCFA production by normal bacteria clearly showed that the BM medium provided the best acetate and propionate productions, although it presented a yield and productivity on propionate similar to the YEL medium. Moreover, we have also observed that the MCHC medium was not appropriate for bacteria growth being unfavorable to produce SCFA. These conditions mimic the real scenario of the gastrointestinal tract (GIT) and therefore indicate that the digestive stress affects the probiotic capacity of propionibacteria, although the production yield showed for acetate is high in comparison to other media. Thus, further developments and optimizations of *P. freudenreichii* for the envisaged use must be explored.

Lan et al. (12) cultured several strains of propionibacteria in MCHC medium at 37°C and evaluated the propionate production. The authors found that a set of strains produced propionate at concentrations around 1.32 g L^−1^, while other strains produced lower amounts, thus suggesting that the propionate production rate is strain-dependent. *P. freudenreichii* subsp. *freudenreichii*, named TL3, produced 1.32 g L^−1^ of propionate, which is similar to the propionate concentration obtained in this study (1.4 g L^−1^). Jan et al. (17) using the same conditions as the ones herein used in the fermentation of DMEM medium showed, with the strain ITG18 (*P. freudenreichii* subsp. *freudenreichii*), that propionibacteria produced 2.7 g L^−1^ of propionate and 0.75 g L^−1^ of acetate. Therefore, the results presented are in accordance with the literature.

In this work, we have successfully adapted *P. freudenreichii* subsp. *freudenreichii* DSM 20271 to the digestive stress by culturing cells in a simulated digestive stress media. Several authors have performed digestive stress challenges to test the adaptation and tolerance of propionibacteria (11, 12, 17, 33). Being probiotics, these bacteria must be able to survive the acid stress imposed within the stomach in order to reach the intestine where they exert their beneficial role. In all the studies reported, the authors could obtain a propionibacteria adapted to the digestive stress, which was able to survive the challenge similarly to our findings. Moreover, these studies showed that the digestive stress tolerance is highly variable, depending on the strain. The strain used in this work is described as the one that presents the best survival rate during digestive stress challenges (12). The adaptation of the *P. freudenreichii* induced a negative impact on the production of propionate in YEL and BM media. However, the performance of adapted *P. freudenreichii* was not affected in the MCHC and DMEM media for which a small increase of biomass production was observed when compared with the results obtained for normal *P. freudenreichii*.

Short chain fatty acids have been widely described in the literature for its variety of biological effects and several reports demonstrate the mechanisms of action of these molecules (19, 21, 22). Both SCFA and propionibacteria culture supernatants from the dairy species *P. freudenreichii* and *P. acidipropionici* induced apoptosis in CRC cells *in vitro* (17, 27, 28, 36). For instance, Lan et al. (27) showed that 0.9 g L^−1^ of acetate and 2.2 g L^−1^ of propionate induced HT 29 colon cancer cells death by 50% at 48 h after incubation. Jan et al. (17) reported that the DMEM fermented medium by *P. freudenreichii* subsp. *freudenreichii* strain ITG18, containing 0.75 g L^−1^ of acetate and 2.7 g L^−1^ of propionate at pH 5.6, was able to induce cell death in HT29 colon cancer cells, and proved that the cytotoxicity was due to the SCFA concentration present in the supernatant. Our results using 0.79 g L^−1^ of acetate and 2.6 g L^−1^ of propionate showed a decrease of cell proliferation by ~21% in RKO cells and an induction of cell-cycle arrest in the G2/M phase, not inducing a significant effect on cell death. This might reflect the different sensitivity to SCFA of the cell lines used, given that these cells have different genetic backgrounds.

To study the potential antineoplastic effect of propionibacteria fermented culture media on CRC cells, cells were treated with DMEM and BM fermented broth by adapted and normal *P. freudenreichii* for 48 h, as described previously. Regarding the cell cycle distribution assessed in the treatments with SCFA and SCFA produced by *P. freudenreichii*, our results showed a cell cycle arrest and proliferation inhibition for all the conditions evaluated (BM and DMEM fermented broth and the respective controls) similarly to what was reported in our own, as well as in others studies (22, 27). However, it is important to highlight that we herein report for the first time the cytotoxic effect of the fermented broth of a propionibacteria medium (BM) on CRC cells.

Cancer cells are known for its biological capabilities acquired during the multistep development of human tumors that makes them act differently from normal cells (37). One of these features is the ability to circumvent the immune system taking advantage of the interaction with stromal cells (37). Also, development of colorectal cancer is related to alterations in the population of the gut microbiota [reviewed in (38)]. Therefore, we evaluated the effect of conditioned medium by CRC cells on the propionibacteria performance, adapted, and non-adapted to digestive stress. The results indicated that propionibacteria grown in conditioned medium provide higher SCFA production when compared with the respective control, being this improvement more pronounced in the acetate production. *P. freudenreichii* may be able to grow in co-culture with CRC cell lines since a slower growth with an increased SCFA production was observed. Moreover, our results demonstrated that this biotransformation performance induced cell death of CRC.

The improvement of SCFAs production in conditioned medium may be due to the metabolites excreted by RKO cell lines, namely lactate. Lactate is an ideal carbon source for *P. freudenrechii* fermentation, leading to high propionate and acetate yields (39). *P. freudenrechii* has a complex metabolism that involves several metabolic pathways. Lactate consumption yields pyruvate which can be reduced to produce propionate via the transcarboxylase cycles, or oxidized to yield acetate and CO_2_ (40).

CRC is known to rely on glycolysis for energy production and this activity leads to the production of important amounts of lactate, which are exported into the extracellular milieu, thus contributing to the acidic microenvironment (41, 42). This phenomenon, known as “aerobic glycolysis” or “Warburg effect” (43) and it leads to an accumulation of lactate (44). The increased lactate production by CRC cells (42) may result, as we observed in this work, in the increased production of SCFA by Propionibacterium. Our results suggest that *P. freudenreichii* promotes a cytotoxic effect on CRC cells, via their metabolites, and that the CRC cells increases the acetate and propionate production by *P. freudenreichii*, which in turn will lead to an increased cytotoxic effect on the CRC cells.

## Conclusions

*Propionibacterium freudenreichii* was able to produce high amounts of acetate and propionate in BM and YEL media. Adaptation of *P. freudenreichii* to digestive stress was successfully obtained and its behavior was similar to the normal bacteria. Interestingly, we observed a great improvement in the bacteria performance using the MCHC and DMEM media which are similar to the real scenario of the GIT. The effect of CRC cells conditioned medium rich in lactate on the bacterial performance showed that bacteria were able to grow remaining metabolically active and produced more SCFA in the presence of lactate. Furthermore, the exposure of CRC cells to SCFA produced by *Propionibacteria* inhibited their proliferation and induced cell cycle arrest. Our results suggest the use of *P. freudenreichii* as a probiotic in the prevention and/or as therapeutic adjuvant in CRC.

## Author contributions

The experimental work was developed by MC. All authors contributed to the planning, discussion, and writing of the manuscript and have given approval to its final version. Both senior authors designed the study, planed the experimental work and contributed equally to this article.

### Conflict of interest statement

The authors declare that the research was conducted in the absence of any commercial or financial relationships that could be construed as a potential conflict of interest.
